# Evidence for Internal Misalignment of Circadian Rhythms in Youth With Emerging Mood Disorders

**DOI:** 10.1177/07487304251349408

**Published:** 2025-07-15

**Authors:** Joanne S. Carpenter, Jacob J. Crouse, Mirim Shin, Emiliana Tonini, Gabrielle Hindmarsh, Zsofi de Haan, Frank Iorfino, Rebecca Robillard, Sharon Naismith, Elizabeth M. Scott, Ian B. Hickie

**Affiliations:** * Youth Mental Health and Technology Team, Brain and Mind Centre, The University of Sydney, Sydney, NSW, Australia; † Sleep Research Unit, University of Ottawa Institute of Mental Health Research at the Royal, Ottawa, ON, Canada

**Keywords:** melatonin, cortisol, core body temperature, depression, bipolar, chronobiology

## Abstract

Despite evidence for links between circadian dysfunction and mood disorders, previous research has largely reported on single biological markers of circadian alignment. The available evidence on relationships between 2 internal phase markers (e.g., dim light melatonin onset [DLMO] and peak cortisol concentration) suggests these signals may be temporally misaligned in major depressive disorder with greater misalignment associated with more severe depressive symptoms. This study aimed to examine multiple circadian phase markers to determine whether any youth with emerging mood disorders present with clear evidence of internal circadian misalignment, and whether the degree of circadian misalignment is correlated with more severe mood symptoms. Cross-sectional data from 69 youth presenting for mental health care (20.6 ± 3.8 years; 39% male) and 19 healthy controls (24.0 ± 3.6 years; 53% male) included actigraphy monitoring; overnight in-lab measurement of 3 phase markers: DLMO, salivary cortisol peak (CORT), and core body temperature nadir (TEMP); and depressive symptoms (Hamilton Depression Rating Scale). Abnormal phase angles between 2 phase markers were defined as ±2 standard deviations beyond the control mean. In those with emerging mood disorders, earlier TEMP relative to other phase markers (DLMO, CORT, sleep midpoint) was associated with higher depressive symptoms. Sixteen individuals (23%) with emerging mood disorders had abnormal phase angles between at least 1 pair of phase markers, consistent with internal misalignment of the circadian system. The internal misalignment subgroup had later DLMO on average, however presented with a diverse range of individual phase angle abnormalities. Diverse disruptions of circadian alignment occur in youth with mental ill-health. The relative timing of core body temperature and melatonin rhythms may be key circadian features linked to depressive symptoms. Longitudinal research is needed to establish whether correction of circadian misalignment is relevant to treatment of mood syndromes in youth with evidence of disrupted circadian systems.

Dysfunction of the 24-h circadian system has been highlighted as a plausible mechanism underlying mood disturbance in a subset of individuals with mood disorders (Carpenter et al., 2021; Crouse et al., 2021; Fishbein et al., 2021; de Leeuw et al., 2023; Meyer et al., 2024). Evidence to support this proposal includes reports of abnormalities in various parameters under circadian influence, including rest-activity (sleep-wake) rhythms, subjective energy and fatigue, sensitivity to seasonal changes, rhythms of hormone secretion (e.g., melatonin, cortisol), rhythms of body temperature (Carpenter et al., 2021; de Leeuw et al., 2023; Hickie et al., 2023; Meyer et al., 2024), as well as evidence suggesting increased cross-domain reactivity among measures of mood, energy, and sleep in some people with mood disorders (which could potentially reflect a failure of circadian homeostasis) (Merikangas et al., 2018). Evidence linking the 24-h circadian system to the onset and/or course of mood disorders spans many areas of investigation, including genetic (Pagani et al., 2015), behavioral (McCarthy et al., 2022; Scott et al., 2022), biological (Jagannath et al., 2013; McGlashan et al., 2019; Carpenter et al., 2021), epidemiologic (Byrne et al., 2019; Kang et al., 2024), and treatment-related (Robillard et al., 2018a; McCarthy et al., 2019; Crouse et al., 2024) studies. However, the nature of these circadian-related abnormalities is not uniform across studies. In particular, there are conflicting reports of biological circadian timing being advanced (i.e., shifted earlier), delayed (i.e., shifted later), or neither in people with mood disorders as compared to healthy controls (Nair et al., 1984; von Zerssen et al., 1985; Souetre et al., 1989; Crasson et al., 2004; Germain and Kupfer, 2008; Robillard et al., 2013; Gonzalez, 2014; Moon et al., 2016). One factor that might contribute to this discrepancy is that the observation of a shifted rhythm could reflect a simple form of circadian disruption caused by misalignment to the light-dark cycle, or alternatively, a shifted rhythm could instead be a marker of a more complex endogenous circadian disruption such as “internal misalignment.” Internal misalignment refers to when multiple circadian-regulated processes, such as melatonin secretion, cortisol release, and core body temperature rhythms, become misaligned with each other (internal alignment), as opposed to being misaligned with the external environment (external alignment). To quantify internal alignment, multiple biological phase markers need to be measured simultaneously, often requiring controlled laboratory conditions to reduce the masking influence of behavioral and environmental factors. Importantly, while such protocols are necessary to probe biological circadian function, the observed effects may not be generalizable to field settings and do not provide information about variability over time (Vetter, 2020). Accordingly, highly controlled laboratory protocols may benefit from concurrent field measures (e.g., actigraphy) to provide supplementary information about real-world behavior (Vetter, 2020).

Most previous studies in mood disorders reporting on biological circadian measures have focused on single-phase markers in relation to external clock time or sleep time, with only a few studies reporting the relationships between multiple internal phase markers. One pilot study of 6 individuals with major depressive disorder (MDD) compared with 6 healthy controls found differences in the timing relationship between peak cortisol concentration and dim light melatonin onset (DLMO), with a longer phase angle (time lag) between these 2 markers in individuals with MDD (Buckley and Schatzberg, 2010). Another study of 9 individuals with MDD reported an increase of the cortisol-melatonin phase angle following treatment with brexpiprazole, which was associated with a reduction in depressive symptoms (Krystal et al., 2021). In a similar vein, while not a direct phase angle comparison, a sample of 11 people with bipolar depression was reported to have a normal cortisol phase, but an abnormal melatonin phase (which shifted following recovery) compared with 10 controls (Fang et al., 2021). In contrast, Hasler and colleagues did not find differences in phase angles of DLMO and core body temperature between 14 individuals with MDD and 13 healthy controls; however, they did report correlations between higher depressive symptoms and phase angles indicative of later relative core body temperature (Hasler et al., 2010). Correlations between higher depressive symptoms and phase angles indicating later DLMO relative to sleep timing have also been reported in a sample of 18 women with MDD (Emens et al., 2009) and a sample of 25 healthy first-year medical students (Emens and Lewy, 2023). A recent study examining wearable-inferred digital markers of circadian disruption in 800+ first-year training physicians reported that a greater misalignment between a modeled estimate of the circadian rhythm of the central oscillator and sleep midpoint was associated with poorer next-day mood and later clinically relevant changes in depressive symptoms (Lee et al., 2024). In clinical samples, we have also previously presented preliminary data describing high variation in individual DLMO-core body temperature phase relationships in 8 young people with unipolar or bipolar mood disorders (Robillard et al., 2013). In addition, we have previously reported that subgroups of young people with mood disorders present with delayed sleep-wake, melatonin, and core body temperature rhythms, but—at the group level—do not significantly differ in melatonin-core body temperature phase angles compared with controls or non-delayed subgroups (Carpenter et al., 2017; Robillard et al., 2018b).

Taken together, the research to date is consistent with the phenomenon of internal circadian misalignment in some individuals with mental ill-health. However, samples are small, and no more than 2 phase markers have been examined in a single study. Furthermore, comparisons have generally been made between mood disorder groups as a whole and healthy controls, which may be hindered by heterogeneity in affective state and circadian function within those with mood disorders. Indeed, Hasler and colleagues reported greater within-group variability of phase angles in the MDD group, and we reported that certain circadian patterns (i.e., delays) only occur in a subgroup of individuals with mood disorders (Carpenter et al., 2017; Robillard et al., 2018b) and that abnormal phase angles are seen when examining individual patterns (Robillard et al., 2013, 2018b).

Accordingly, the present study aims to examine the presence and degree of circadian misalignment across multiple phase markers in a larger sample of young people with emerging mood disorders presenting for mental health care. We aimed to examine whether phase angles between DLMO, core body temperature nadir, cortisol peak, and sleep timing are significantly associated with depressive symptoms. We further sought to determine whether youth with abnormal internal phase angles differ in clinical presentation from those with normal circadian alignment. Based on previous research outlined above, we hypothesized that (1) in individuals with emerging mood disorders, increased depressive symptoms would be significantly correlated with greater misalignment in the direction of later relative melatonin or core body temperature rhythms (i.e., shorter phase angles between melatonin and cortisol, melatonin and sleep midpoint, core body temperature and cortisol, and core body temperature and sleep midpoint); and (2) a subgroup of youth with emerging mood disorders would present with abnormal phase angles (i.e., internal circadian misalignment), likely driven by delays in melatonin or core body temperature timing relative to cortisol.

## Methods

### Participants

Participants were young people (aged 16-35 years) presenting for mental health care at primary care–based early intervention mental health services in Sydney, Australia (Scott et al., 2012). Healthy control participants with no history of a mental disorder were recruited from the local community. Participants were recruited between November 2012 and September 2014. Participants were excluded on the basis of intellectual disability, insufficient English for clinical assessment, mental health syndromes secondary to medical conditions or substance dependence, neurological conditions (e.g., epilepsy), sleep disorders (e.g., sleep apnoea), current severe suicidality, and current use of hypnotics, benzodiazepines, or melatonin-based medication. No participant reported regular shiftwork or transmeridian travel within 60 days prior to study commencement. The research was approved by the University of Sydney Human Research Ethics Committee (02-2008/11445) and all participants gave written informed consent.

Some analyses of melatonin and core body temperature rhythms have been reported previously in this sample (Carpenter et al., 2017; Robillard et al., 2018b); however, this is the first analysis of 3 phase markers in tandem. Participants were included in the current analysis if they had at least 2 available measures from assessments of DLMO, waking cortisol, and core body temperature. A flow diagram of inclusion and exclusion is presented in Supplementary Figure S1.

### Clinical Assessment

For participants with emerging mood disorders, semi-structured interviews were conducted by a psychiatrist or research psychologist, which included administration of the Hamilton Depression Rating Scale (HDRS) (Hamilton, 1967), Young Mania Rating Scale (YMRS) (Young et al., 1978), and Social and Occupational Functioning Assessment Scale (SOFAS) (Goldman et al., 1992). These measures were also available for a subset of controls (*n* = 8).

Primary diagnosis was assigned from the best available information which included the Brain and Mind Centre Optymise Cohort clinical proforma (Carpenter et al., 2020), reports from referring clinicians, and semi-structured interviews by a psychiatrist or research psychologist. Primary diagnoses were grouped into 4 broad categories as follows: depressive disorder (*n* = 34), bipolar disorder (*n* = 5), anxiety disorder (*n* = 15), and other (psychotic disorder, *n* = 2; neurodevelopmental disorder, *n* = 3; obsessive compulsive disorder, *n* = 1; post-traumatic stress disorder, *n* = 1). Diagnosis was missing for 8 individuals.

Medication information was collected via self-report and clinical interview. Medication information was missing for 2 individuals. Of the remaining individuals with emerging mood disorders, 38 (57%) were not taking any psychotropic medications, 22 (33%) were taking 1 class of psychotropic medication, and 6 (9%) were taking 2 classes of psychotropic medication. Specifically, 18 (27%) were taking a selective serotonin reuptake inhibitor(s) (SSRI), 7 (10%) were taking a serotonin and norepinephrine reuptake inhibitor(s) (SNRI), 7 (10%) were taking a mood stabilizer(s), 1 (1%) was taking a stimulant(s), and 1 (1%) was taking an antipsychotic(s).

### Sleep-Wake Assessment

Participants wore a wrist mounted actigraphy recording device (Actiwatch-64/L/2/Spectrum; Philips Respironics, Pittsburgh, USA; or GENEActiv; Activinsights, Kimbolton, UK) for between 5 and 22 days (median 13 days) prior to the in-lab circadian assessment. Participants were instructed to wear the device on their non-dominant wrist and to only remove it for showering, bathing, or swimming. Actiwatch data were collected over 30-sec or 1-min epochs; GENEActiv data were collected at 30 or 50 Hz. All sets of data were visually inspected by a trained technician (JSC/RR) to determine estimates of the start and end of each sleep episode based on the respective drop and rise in motor activity. Additional information was used to inform these judgments where available (i.e., sleep diaries, event markers on the device pressed at bed and rise times, and/or ambient light from sensors on some devices). Sleep onset and offset time were averaged across the recording period. Sleep midpoint was calculated as the halfway point between sleep onset and sleep offset. Due to equipment failure, actigraphy data were missing for 1 participant. Visual inspection of actigraphy data identified 4 individuals with “drifting” sleep-wake patterns indicative of potential non-24-h sleep-wake disorder (all from the emerging mood disorder group)- these individuals were excluded from analyses due to likely inaccuracy of phase measurements based on a single recording (see Supplementary Figure S2 and S3).

### Circadian Assessment

Within 30 days of the sleep-wake (actigraphy) assessment (range 0-27 days; mean = 5.7 days; standard deviation = 5.4 days), participants attended the Brain and Mind Centre sleep laboratory for an overnight circadian assessment based on a semi-constant routine protocol (91% attended within 2 weeks, 68% attended within 1 week). Supplementary Table S1 reports the day of week of the circadian assessment by control and mood disorder groups. On the day of the circadian assessment, participants were instructed not to consume caffeine from 12 p.m. onward. The assessment began approximately 8 h prior to the participant’s habitual sleep time (i.e., average sleep onset time across the actigraphy recording period). They were kept in dim light (<30 lux) while remaining seated as much as possible (participants were permitted to use the bathroom immediately following collection of a sample, but were asked to remain seated otherwise) until approximately 2 h after their habitual sleep time, at which point they were permitted to sleep until their habitual wake time (i.e., average sleep offset time across the actigraphy recording period). At their habitual wake time, they returned to a seated position in dim light for a further 2.5 h. The dim light level threshold (<30 lux) is lower than current recommendations (<10 lux) (St Hilaire and Lockley, 2022); however, the protocol was conducted during 2012-2014 and designed according to a 2008 consensus guideline that recommended a <30 lux threshold for DLMO studies (Benloucif et al., 2008).

Saliva samples were collected with Salivette tubes (Sarstedt, Nümbrecht, Germany) for measurement of melatonin and cortisol. Samples for melatonin were collected every 30 min from approximately 6 h prior to habitual sleep time until approximately 2 h after habitual sleep time. To capture an estimate of cortisol peak while balancing participant burden in light of other protocol demands, 3 cortisol samples were collected: hourly from 15 min after habitual wake time until 2.25 h after habitual wake time.

An ingestible sensor (Equivital LifeMonitor; Equivital, Cambridge, UK) was used to record core body temperature from shortly after arrival at the laboratory until approximately 3 h after habitual wake time. Participants were provided with temperature-controlled snacks and drinks to minimize disruption of the temperature recording.

Hormonal assays were conducted at the University of Adelaide Research Assay Facility. Salivary melatonin was assayed in duplicate by double antibody radioimmunoassay (Buhlmann Laboratories AG, Schanenbuch, Switzerland) with a detection threshold of 0.999 pg/mL (inter-assay coefficient of variation between 8.2% and 22.4%). DLMO was estimated using the hockey-stick method with Hockey-stick v2.5 software (Danilenko et al., 2014; Glacet et al., 2023). In 3 of the 4 participants with emerging mood disorders and non-24-h sleep-wake patterns, DLMO could not be estimated due to high melatonin concentration from the first available sample; for the remaining participant, melatonin only rose above the detectable threshold for the final sample (see Supplementary Figure S3). DLMO could not be estimated in an additional 7 individuals with emerging mood disorders as the hockey-stick algorithm could not detect a dynamic part of the melatonin profile (see Supplementary Figure S4), 2 of these individuals were also missing temperature nadir time due to insufficient data (see below) and were not included in either mood disorder group for comparative analyses. As an alternative method of calculating DLMO, a threshold defined by linear interpolation based on the 2 samples surrounding the sample where salivary melatonin concentration reached a threshold of 3 pg/mL and remained above this threshold for the 3 subsequent samples was used in supplementary analyses (see below).

Salivary cortisol was assayed in duplicate by enzyme-linked immunosorbent assay (ELISA) (Salimetrics, State College, PA, USA), with a detection threshold of 1.0 nM (inter-assay coefficient of variation between 5.3% and 14.6%). An estimate of the timing of the cortisol peak was taken as the time when the sample with the highest value occurred. Cortisol peak was not calculated in 6 participants with emerging mood disorders due to one or more missing samples.

Temperature recordings were taken several times per minute and averaged to 1-min epochs across the recording period. The first hour of recording was discarded to remove the initial variation related to gastrointestinal transit. Any epoch in which temperature differed by more than 0.15 °C from the previous minute was considered a non-physiological change in temperature and was discarded along with the following 30 min in which temperature returned to normal. Curve fitting was performed using GraphPad Prism (GraphPad Software, La Jolla, CA, USA) to fit a sine wave to each participant’s temperature recording. The sine wave was restricted to an absolute frequency of <0.5 (no more than 1 cycle per 12 h) to best fit the available portion of the curve (as recordings were less than 24 h in duration) (Minors and Waterhouse, 1988). From the best curve fit, the core temperature minimum (lowest temperature) and nadir (time at which the minimum occurred) were estimated. Curve fitting could not be performed in 19 participants with emerging mood disorders and 4 controls due to insufficient data (i.e., recordings terminated early more than 1.5 h before habitual wake time or there was less than 9 h total data resulting in ambiguous curve fits).

Phase angles were calculated as the time difference between 2 phase markers for: DLMO and core temperature nadir (DLMO-Temp calculated as core temperature nadir time minus DLMO time), DLMO and cortisol peak (DLMO-Cortisol calculated as cortisol peak time minus DLMO time), core temperature nadir and cortisol peak (Temp-Cortisol calculated as cortisol peak time minus core temperature nadir time), DLMO and sleep midpoint (DLMO-Sleep calculated as sleep midpoint time minus DLMO time), core temperature nadir and sleep midpoint (Temp-Sleep calculated as sleep midpoint time minus core temperature nadir time), and cortisol peak and sleep midpoint (Sleep-Cortisol calculated as cortisol peak time minus sleep midpoint time). Prior to calculation of phase angles, the integer 24 was added to any time value between 0 and 16 (i.e., midnight and 4 p.m.) to ensure a continuous scale between measures.

Height and weight were measured for calculation of body mass index (BMI) using the formula weight(kg) ÷ height(m)^2^. BMI data were missing for 6 individuals with emerging mood disorders.

### Statistical Analysis

Analyses were performed using R (version 4.0.5) in RStudio (version 2023.12.1). A *p* value of <0.05 was considered statistically significant. Pairwise deletion was used for missing data. Multiple linear regression was used to examine relationships between circadian measures and clinical measures controlling for age and sex at birth. As exploratory supplementary analysis, multiple linear regression further examined relationships between circadian measures and individual items of the HDRS (Supplementary Table S2 and Figure S5). Participants with internal circadian misalignment (i.e., abnormal phase angles) were operationalized as those who had one or more of the following phase angles outside the mean ± 2SD of controls: DLMO-Temp, DLMO-Cortisol, Temp-Cortisol. This cutoff was chosen to identify individuals with circadian alignment at the extreme ends of a normal distribution. This resulted in 4 groups: controls with typical internal circadian alignment (controls-circadian aligned), individuals with emerging mood disorders and typical internal circadian alignment (mood disorder-circadian aligned), individuals with emerging mood disorders and internal circadian misalignment (mood disorder-circadian misaligned), and controls with internal circadian misalignment (control-circadian misaligned). As there was only one individual in the control-circadian misaligned group, this group is presented in tables for descriptive purposes only and not included in group comparisons. For the following analyses, distributions of all dependant variables were inspected for normality using QQ plots and Shapiro-Wilk tests and any non-normally distributed variables were transformed with Box-Cox transformation prior to analysis. Analysis of variance (ANOVA) was used to compare groups across demographic variables; analysis of covariance (ANCOVA) was used to compare the groups across sleep-wake and circadian measures, controlling for age and sex at birth. For significant ANOVA/ANCOVA models, Tukey’s test was used to identify significant pairwise differences. ANCOVA was used to compare clinical measures between the mood disorder-circadian aligned and mood disorder-circadian misaligned groups controlling for age and sex at birth. χ^2^ tests were used to compare primary diagnoses and medication use between the mood disorder-circadian aligned and mood disorder-circadian misaligned groups. Finally, analyses were repeated using an alternative DLMO calculation method with a 3 pg/mL threshold as described above (see Supplementary Tables S4 and S5).

## Results

### Sample Characteristics

**Figure 1.**
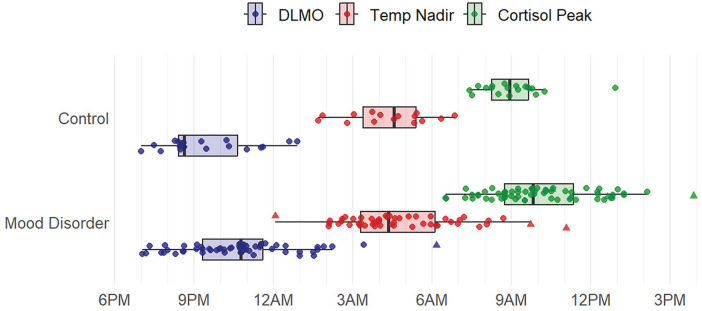
Timing of DLMO, core body temperature nadir, and cortisol peak in controls and youth with emerging mood disorders. Abbreviations: DLMO = dim light melatonin onset, Temp Nadir = Core Body Temperature Nadir. Boxplots display the median and interquartile range, dots represent individual data points, triangles represent individuals with non-24-h actigraphy patterns.

**Figure 2.**
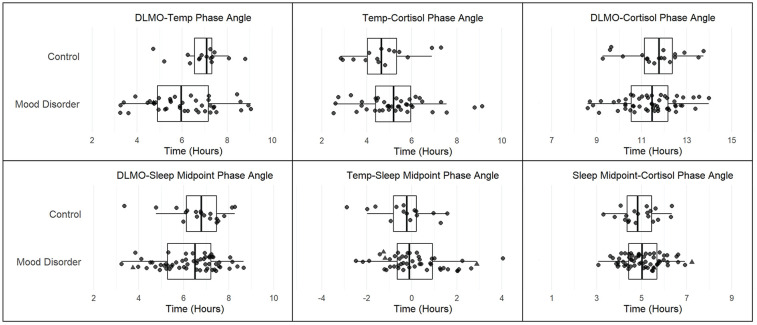
Distribution of phase angle differences in controls and youth with emerging mood disorders. Abbreviations: DLMO = dim light melatonin onset; Temp = Core Body Temperature Nadir. Boxplots display the median and interquartile range, dots represent individual data points, triangles represent individuals with non-24-h actigraphy patterns.

A total of 69 individuals with emerging mood disorders (mean age = 20.6 ± 3.8, 39% male) and 19 healthy controls (mean age 24.0 ± 3.6, 53% male) were included in the final sample. Table 1 reports demographic and clinical features of controls and individuals with emerging mood disorders. The mood disorder group had a mean HDRS of 15.1 ± 6.0, YMRS of 3.2 ± 3.9, and SOFAS of 63.0 ± 8.6, indicating mild-moderate depressive symptoms, subthreshold hypomania symptoms, and mild impairment in functioning on average. The distribution of phase markers in controls and people with emerging mood disorders is displayed in Figure 1, and distributions of phase angles are displayed in Figure 2. Four individuals with mood disorders and drifting sleep-wake patterns (potential non-24-h patterns) were excluded from further comparative analyses (see Supplementary Materials).

**Table 1.** Demographic and clinical features of controls and youth with emerging mood disorders.

	Controls	Youth With Emerging Mood Disorders
N	19	69
Age (years)	23.95 (3.60)	20.64 (3.80)
Sex (M/F)	10/9	27/42
BMI	23.13 (3.46)	24.66 (5.34)
Sleep Onset time	0:07 (0:52)	0:45 (1:51)
Sleep Offset time	8:13 (1:09)	9:26 (1:49)
Sleep Midpoint time	4:10 (0:56)	5:06 (1:46)
Sleep Duration (min)	436.65 (50.48)	444.73 (52.71)
Wake After Sleep Onset (min)	47.83 (11.88)	76.39 (28.76)
DLMO time	21:30 (1:44)	22:46 (2:08)
Cortisol Peak time	9:04 (1:15)	10:07 (1:59)
Core Temperature Nadir time	4:20 (1:32)	4:48 (2:10)
SOFAS	84.38 (7.09)^a^	62.95 (8.58)
YMRS	0.88 (1.25)^a^	3.15 (3.92)
HDRS	2.25 (1.67)^a^	15.09 (6.04)
Any Psychiatric Medication, *N* (%)	0 (0%)	29 (43%)

Values represent mean (standard deviation) unless specified otherwise.

Abbreviations: BMI = body mass index; DLMO = dim light melatonin onset; SOFAS = Social and Occupational Functioning Assessment Scale; YMRS = Young Mania Rating Scale; HDRS = Hamilton Depression Rating Scale.

a Measures that were only available in a subset of controls (*n* = 8).

### Associations Between Circadian Measures and Symptom and Functioning Measures in Youth With Emerging Mood Disorders

**Figure 3.**
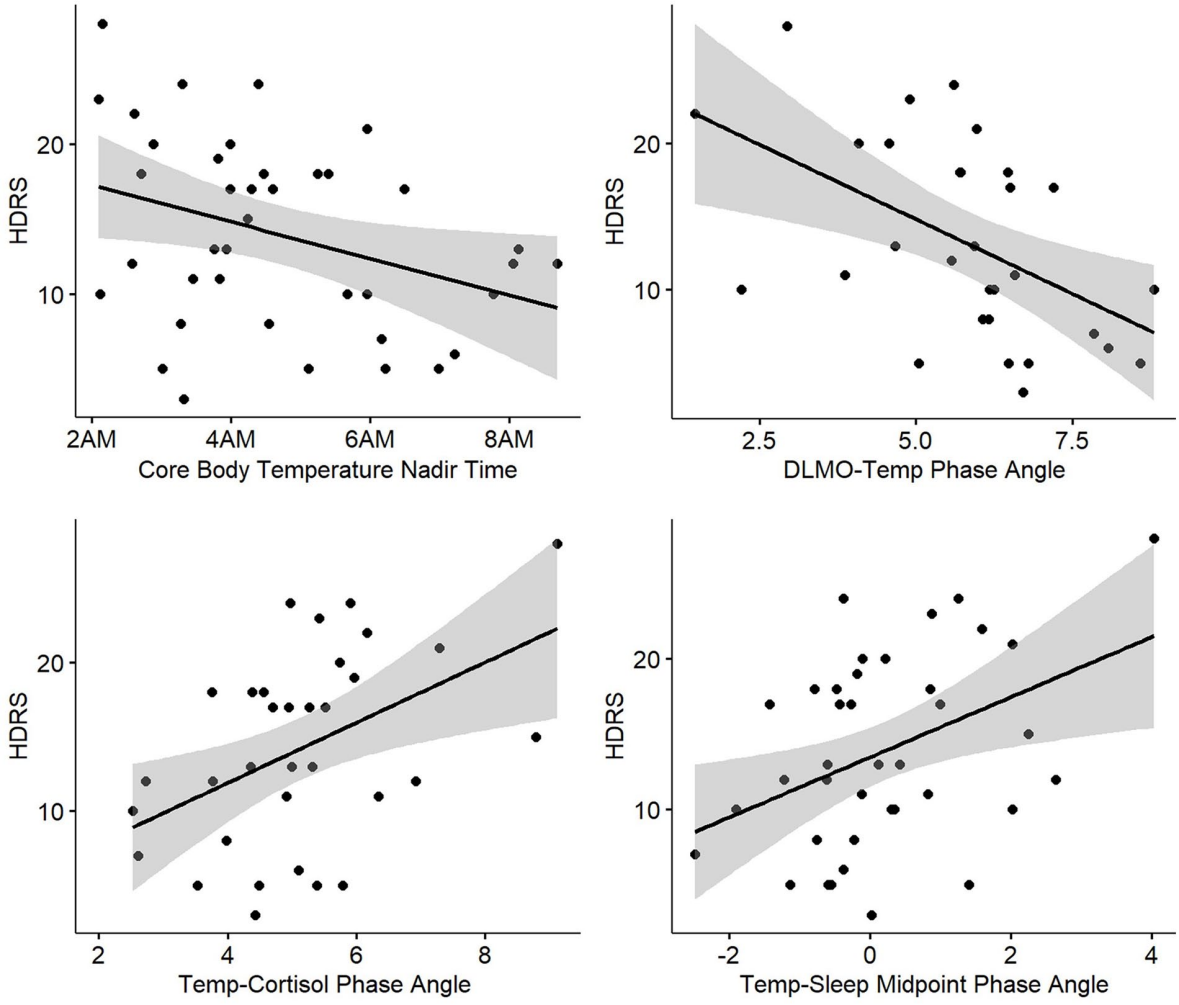
Scatterplots of significant correlations between circadian measures and depressive symptoms in youth with emerging mood disorders. Abbreviations: DLMO = dim light melatonin onset; HDRS = Hamilton Depression Rating Scale; Temp = Core Body Temperature Nadir.

Associations between circadian measures and symptom and functioning measures within the mood disorder group are reported in Table 2, with scatterplots for significant correlations in Figure 3. Higher depressive symptoms (HDRS) were significantly associated with earlier core body temperature nadir (*B* = −1.27; *p* = 0.03), shorter DLMO-Temp (*B* = −2.18; *p* < 0.01), longer Temp-Cortisol (*B* = 2.22; *p* < 0.01), and Temp-Sleep Midpoint (*B* = 1.95; *p* = 0.02) phase angles indicating earlier timing of core body temperature nadir relative to external clock time, DLMO, cortisol peak, and sleep midpoint. Exploratory supplementary analysis examining relationships between circadian measures and individual items of the HDRS found significant associations mostly with symptoms of insomnia (middle or late), impairment in work and activities, anxiety, and somatic symptoms (Supplementary Table S2 and Figure S5).

**Table 2.** Associations between circadian and symptom and functioning measures in youth with emerging mood disorders controlling for age and sex at birth.

Outcome Variable	Predictor Variable	Intercept	*F*-statistic	Slope (B)	t-Value	*p*-Value
SOFAS	DLMO Time	65.54	*F*_(3,48)_ = 2.19, *p* = 0.101	0.02	0.02	0.98
	Cortisol Peak Time	65.48	*F*_(3,50)_ = 2.15, *p* = 0.105	-0.79	-1.17	0.25
	Core Body Temperature Nadir Time	67.27	*F*_(3,38)_ = 4.8, *p* = 0.006	-1.3	-1.78	0.08
	Sleep Midpoint	65.18	*F*_(3,55)_ = 1.91, *p* = 0.138	-0.19	-0.24	0.81
	DLMO-Temp Phase Angle	68.08	*F*_(3,32)_ = 3.91, *p* = 0.018	-0.03	-0.03	0.98
	Temp-Cortisol Phase Angle	68.15	*F*_(3,32)_ = 3.62, *p* = 0.023	-0.63	-0.69	0.50
	DLMO-Cortisol Phase Angle	65.77	*F*_(3,43)_ = 2.81, *p* = 0.051	-1.31	-1.27	0.21
	DLMO-Sleep Midpoint Phase Angle	65.62	*F*_(3,48)_ = 2.24, *p* = 0.096	0.36	0.35	0.73
	Temp-Sleep Midpoint Phase Angle	67.51	*F*_(3,37)_ = 3.58, *p* = 0.023	0.25	0.27	0.79
	Sleep Midpoint-Cortisol Phase Angle	65.88	*F*_(3,50)_ = 3.07, *p* = 0.036	2.8	1.97	0.05
YMRS	DLMO Time	2.85	*F*_(3,42)_ = 0.6, *p* = 0.619	-0.29	-0.93	0.36
	Cortisol Peak Time	2.65	*F*_(3,42)_ = 0.9, *p* = 0.449	-0.34	-1.06	0.29
	Core Body Temperature Nadir Time	2.58	*F*_(3,32)_ = 0.2, *p* = 0.894	0.01	0.03	0.98
	Sleep Midpoint	2.72	*F*_(3,47)_ = 0.62, *p* = 0.603	-0.2	-0.5	0.62
	DLMO-Temp Phase Angle	2.63	*F*_(3,27)_ = 0.11, *p* = 0.954	0.01	0.03	0.97
	Temp-Cortisol Phase Angle	2.36	*F*_(3,26)_ = 0.24, *p* = 0.870	-0.32	-0.66	0.52
	DLMO-Cortisol Phase Angle	2.64	*F*_(3,37)_ = 0.51, *p* = 0.676	-0.32	-0.62	0.54
	DLMO-Sleep Midpoint Phase Angle	2.85	*F*_(3,42)_ = 0.34, *p* = 0.795	0.18	0.34	0.74
	Temp-Sleep Midpoint Phase Angle	2.53	*F*_(3,31)_ = 0.36, *p* = 0.782	-0.36	-0.69	0.50
	Sleep Midpoint-Cortisol Phase Angle	2.88	*F*_(3,42)_ = 1.41, *p* = 0.253	1.18	1.62	0.11
HDRS	DLMO Time	13.89	*F*_(3,44)_ = 1.24, *p* = 0.308	-0.15	-0.32	0.75
	Cortisol Peak Time	14.1	*F*_(3,45)_ = 1.07, *p* = 0.373	-0.26	-0.54	0.59
	Core Body Temperature Nadir Time	13.11	*F*_(3,35)_ = 2.18, *p* = 0.107	-1.27	-2.31	0.03*
	Sleep Midpoint	13.89	*F*_(3,50)_ = 1.12, *p* = 0.35	-0.54	-0.98	0.33
	DLMO-Temp Phase Angle	12.83	*F*_(3,29)_ = 4.47, *p* = 0.011	-2.18	-3.4	<.01**
	Temp-Cortisol Phase Angle	13.12	*F*_(3,29)_ = 3.55, *p* = 0.027	2.22	3.06	<.01**
	DLMO-Cortisol Phase Angle	13.96	*F*_(3,39)_ = 1.75, *p* = 0.172	-0.53	-0.71	0.48
	DLMO-Sleep Midpoint Phase Angle	13.59	*F*_(3,44)_ = 1.65, *p* = 0.193	-0.83	-1.11	0.27
	Temp-Sleep Midpoint Phase Angle	13.3	*F*_(3,34)_ = 2.47, *p* = 0.078	1.95	2.53	0.02*
	Sleep Midpoint-Cortisol Phase Angle	13.88	*F*_(3,45)_ = 1.12, *p* = 0.350	-0.75	-0.67	0.51

Abbreviations: SOFAS = Social and Occupational Functioning Assessment Scale; YMRS = Young Mania Rating Scale; HDRS = Hamilton Depression Rating Scale; DLMO = dim light melatonin onset; Temp = Core Body Temperature Nadir. Multiple linear regression models including age and sex as covariates. Predictor variables were centered across all youth with emerging mood disorders and female was the reference category for sex. Slopes represent unstandardized regression coefficients.

* *p* < 0.05 and **p < 0.01 in *t*-test.

### Circadian Misalignment in Emerging Mood Disorders: Prevalence and Group Comparisons

**Figure 4.**
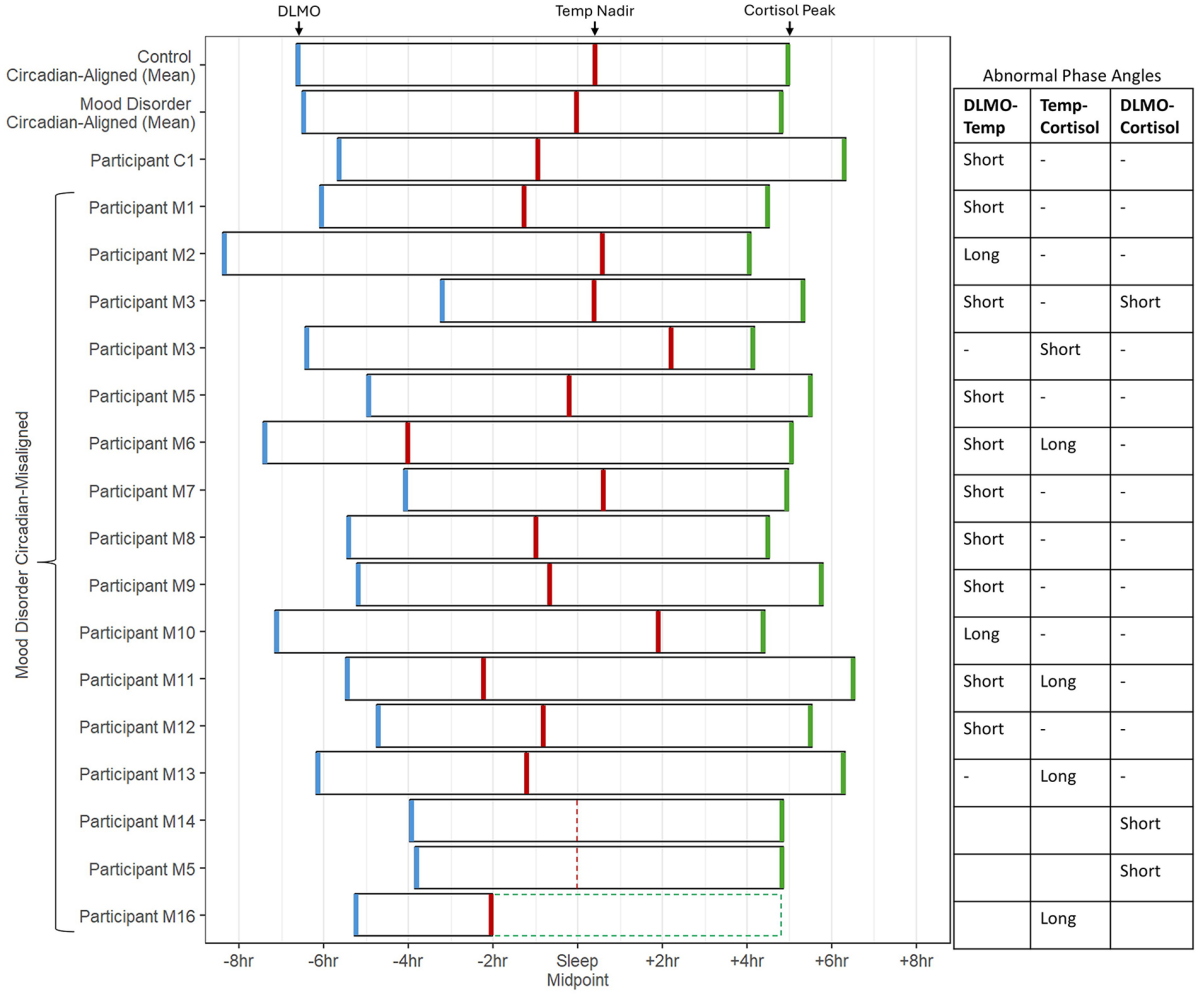
Timing of phase markers in youth with emerging mood disorders and internal circadian misalignment. Boxes and colored lines represent the timing of DLMO (blue), Core Temperature Nadir (red), and Cortisol Peak (green) relative to sleep midpoint. Dotted lines indicate missing data; for illustrative purposes, we have interpolated the mean for these missing values with the average of the mood disorder group. The mean timing for controls with normal circadian alignment (controls-circadian aligned), and individuals with emerging mood disorders and normal internal circadian alignment (mood disorder-circadian aligned) is displayed alongside individual data for those with internal circadian misalignment. C1 indicates a control participant with internal circadian misalignment. Participant M13 was missing actigraphy data for sleep midpoint estimation and was instead centered on the Mood Disorder group mean. Participants M14 and M15 did not have core body temperature nadir due to missing data. Participant M16 did not have cortisol peak due to missing data.

Sixteen individuals with emerging mood disorders (23% of the total mood disorder group) and 1 control participant (5% of the control group) presented with internal circadian misalignment, defined as one or more internal phase angles outside the mean ± 2 SD of controls. Comparisons of demographic, circadian, sleep, and clinical measures between controls with normal internal circadian alignment (controls-circadian aligned), individuals with emerging mood disorders and normal internal circadian alignment (mood disorder-circadian aligned), and individuals with emerging mood disorders and internal circadian misalignment (mood disorder-circadian misaligned) controlling for age and sex are reported in Table 3. The control-circadian misaligned group is also presented for descriptive purposes, but not included in group comparisons. The mood disorder-circadian aligned group was significantly younger than the control-circadian aligned group (*p* < 0.01). The mood disorder-circadian misaligned group had significantly later DLMO time (*p* < 0.01) compared with the control-circadian aligned group, shorter DLMO-Cortisol phase angles (*p* = 0.03) compared with the mood disorder-circadian aligned group, and shorter DLMO-Temp (*p* < 0.01, *p* = 0.01) and DLMO-midsleep (*p* = 0.02, *p* = 0.01) phase angles compared with the control-circadian aligned and mood disorder-circadian aligned groups, respectively. Groups did not significantly differ on any other clinical or circadian measures. Figure 4 depicts phase angles in individuals in the circadian misaligned groups as compared to the mean of control-circadian aligned and mood disorder-circadian aligned groups.

**Table 3.** Comparisons of demographic, sleep, circadian, and clinical measures between groups.

		Group Mean (SD)					Pairwise comparisons (Tukey HSD, *p* values)		
		Controls Circadian Aligned	Mood Disorder Circadian Aligned	Mood Disorder Circadian Misaligned	Control Circadian Misaligned^a^	ANOVA/ANCOVA/χ^2^	Control Aligned v Mood Disorder Aligned	Control Aligned v Mood Disorder Misaligned	Mood Disorder Aligned v Mood Disorder Misaligned
	N	18	47	16	1				
**Demographics**	Age (years)	23.72 (3.56)	20.26 (3.63)	21.19 (2.86)	28	*F*_(2,78)_ = 6.46, *p* = 0.003	<0.01**	0.09	0.62
	Sex (M/F)	10/8	16/31	7/9	0/1	χ^2^_(2)_ = 2.57, *p* = 0.277	-	-	-
	BMI	23.29 (3.49)	24.47 (5.80)	26.01 (4.59)	20.32	*F*_(2,70)_ = 2.3, *p* = 0.108	0.25	0.1	0.68
**In-Lab Circadian Measures**	DLMO time	21:19 (1:34)	22:18 (1:55)	23:29 (1:37)	0:55	*F*_(2,71)_ = 5.68, *p* = 0.005	0.25	<0.01**	0.06
	Cortisol Peak time	8:52 (0:52)	9:51 (1:59)	10:09 (1:28)	12:55	*F*_(2,71)_ = 1.65, *p* = 0.199	0.51	0.17	0.55
	Core Temperature Nadir time	4:15 (1:34)	4:46 (1:38)	4:23 (2:04)	5:38	*F*_(2,56)_ = 0.44, *p* = 0.647	0.69	0.98	0.77
**Actigraphy Measures**	Sleep Midpoint time	4:01 (0:44)	4:52 (1:37)	4:59 (1:19)	6:35	*F*_(2,75)_ = 1.85, *p* = 0.164	0.24	0.18	0.88
	Sleep Duration (minutes)	434.13 (50.85)	441.09 (55.56)	451.69 (32.86)	479.54	*F*_(2,74)_ = 0.44, *p* = 0.643	0.97	0.83	0.61
**Internal Phase Angles**	DLMO-Temp Phase Angle	7:03 (0:50)	6:29 (0:57)	5:10 (2:06)	4:43	*F*_(2,51)_ = 7.36, *p* = 0.002	0.63	<0.01**	<0.01**
	Temp-Cortisol Phase Angle	4:34 (1:07)	4:56 (1:07)	5:35 (2:10)	7:17	*F*_(2,50)_ = 1.56, *p* = 0.22	0.96	0.28	0.27
	DLMO-Cortisol Phase Angle	11:33 (1:17)	11:36 (1:06)	10:37 (1:24)	12:01	*F*_(2,66)_ = 3.81, *p* = 0.027	0.98	0.06	0.03*
**Circadian/Sleep Phase Angles**	DLMO-Sleep Midpoint Phase Angle	6:43 (1:11)	6:35 (1:02)	5:28 (1:26)	5:41	*F*_(2,70)_ = 5.32, *p* = 0.007	0.92	0.02*	0.01*
	Temp-Sleep Midpoint Phase Angle	-0:25 (1:11)	0:02 (1:07)	0:31 (1:43)	0:58	*F*_(2,54)_ = 1.45, *p* = 0.243	0.71	0.22	0.48
	Sleep Midpoint-Cortisol Phase Angle	4:50 (0:46)	5:01 (0:52)	5:01 (0:41)	6:20	*F*_(2,70)_ = 0.1, *p* = 0.907	0.95	0.9	0.97
**Clinical Measures**	Primary Diagnosis (Dep/Bip/Anx/Other)	-	21/5/9/7	10/0/4/0	-	χ^2^_(3)_ = 5.1, *p* = 0.164	-	-	-
	SOFAS	-	62.93 (8.42)	64.14 (9.68)	-	*F*_(1,53)_ = 0.47, *p* = 0.495	-	-	-
	YMRS	-	3.82 (4.43)	1.25 (1.06)	-	*F*_(1,46)_ = 3.32, *p* = 0.075	-	-	-
	HDRS	-	14.63 (5.95)	15.17 (6.39)	-	*F*_(1,49)_ = 0.2, *p* = 0.661	-	-	-
**Medications**	Any Psychiatric Medication, *N* (%)	-	23 (51%)	10 (63%)	-	χ^2^_(1)_ = 0.24, *p* = 0.622	-	-	-
	SSRI, *N* (%)	-	14 (31%)	3 (19%)	-	χ^2^_(1)_ = 0.39, *p* = 0.534	-	-	-
	SNRI, *N* (%)	-	4 (9%)	3 (19%)	-	χ^2^_(1)_ = 0.37, *p* = 0.544	-	-	-
	Mood, *N* (%)	-	5 (11%)	2 (13%)	-	χ^2^_(1)_ = 0, *p* = 1	-	-	-
	Stimulant, *N* (%)	-	1 (2%)	0	-	χ^2^_(1)_ = 0, *p* = 1	-	-	-
	Antipsychotic, *N* (%)	-	1 (2%)	0	-	χ^2^_(1)_ = 0, *p* = 1	-	-	-

Abbreviations: SD = standard deviation; BMI = body mass index; DLMO = dim light melatonin onset; SOFAS = Social and Occupational Functioning Assessment Scale; YMRS = Young Mania Rating Scale; HDRS = Hamilton Depression Rating Scale; SSRI = selective serotonin reuptake inhibitor; SNRI = serotonin and norepinephrine reuptake inhibitor; ANOVA = analysis of variance (for demographic variables only); ANCOVA = analysis of covariance controlling for age and sex (for all other continuous variables); phase angles and time variables are presented as hours and minutes.

a Control Circadian Misaligned group is included for descriptive purposes but not included in comparative statistics due to small *N* (*N* = 1)

* *p* < 0.05 and **p < 0.01 in pairwise tests.

## Discussion

This study reports a novel examination of the alignment of circadian rhythms in young people presenting for mental health care with emerging mood disorders. Two key findings emerged. First, in the overall mood disorder sample, significant associations were found between increased depressive symptoms and phase angles consistent with earlier relative core body temperature rhythm timing (relative to clock time, melatonin and cortisol rhythms, and sleep midpoint). Second, a subgroup including approximately 23% of youth with emerging mood disorders (and 5% of healthy controls) presented with a profile of internal circadian misalignment where one or more relationships between melatonin, cortisol, and core body temperature rhythms was abnormal. This internal circadian misalignment group had later relative DLMO timing overall (relative to clock time, core temperature and cortisol rhythms, and sleep midpoint), but was not distinguished by any other examined clinical or demographic features.

The present findings build on previous preliminary reports of correlations between phase angles and depressive symptoms (Emens et al., 2009; Hasler et al., 2010). These previous reports found that *delayed* (relative) melatonin or temperature rhythms were associated with increased symptoms, in partial contrast to our findings, which overall are consistent with *advanced* relative core temperature being associated with increased symptoms. However, we do note that our strongest correlation was found for the DLMO-Temp Phase Angle which may be driven by either earlier relative temperature or later relative DLMO timing (which is also consistent with our finding of later relative DLMO timing overall in the circadian misalignment group). We also did not find a significant correlation between depressive symptoms and the DLMO-Sleep Midpoint phase angle, which is consistent with one previous report (Hasler et al., 2010) but in contrast to another (Emens et al., 2009). Differences in age ranges, clinical presentations (with the present sample including transdiagnostic mood presentations), and small sample sizes in the previous work may have contributed to these discrepancies. In addition, it is likely that disruption of internal circadian alignment (and associated depressive symptoms) may occur with deviation from normal in multiple directions. In support of this, a previous study in individuals with seasonal affective disorder reported opposite correlations between phase angles and depressive symptoms in groups that were phase delayed as opposed to phase advanced (Lewy et al., 2006), and we have previously reported increased depressive symptoms in a phase-delayed subgroup of youth with mood disorders (partially overlapping with the present sample) (Robillard et al., 2018b).

Interestingly, we did not find any significant correlations between phase angles and (hypo)manic symptoms or social and occupational functioning (which, to our knowledge, has not been examined previously). This may suggest that circadian misalignment is more closely linked to depressive phenomenology; however, we note that (hypo)manic symptoms were low overall, and that we have previously reported that circadian abnormalities may be more prominent in those with bipolar-type illness (Robillard et al., 2013). It is important for future work to compare circadian alignment across different phases of illness to better understand the potential role of misalignment in both depressive and manic phenomena.

To further the point that circadian misalignment may present in heterogeneous ways, the identified subgroup of individuals with emerging mood disorders and internal circadian misalignment did not present with uniform phase angle abnormalities. While this group had later relative DLMO timing overall, individual patterns of short and long phase angles were varied (Figure 4). This highlights the inadequacy of group-mean comparisons between heterogeneous groups, emphasizes the importance of examining multiple phase markers, and demonstrates diverse circadian abnormalities which may occur in young people with mental ill-health. We also identified one individual from the control group with circadian misalignment, and note that heterogeneity in circadian alignment is not exclusive to mood disorder samples with wide ranges in phase and alignment reported more broadly in healthy samples (Burgess and Fogg, 2008; Kennaway, 2023). The internal circadian misalignment group did not significantly differ from the rest of the mood disorder group on any clinical measures, suggesting that there is not a simple relationship with cross-sectional measures of severity. Future examination of subtypes of circadian misalignment may also be required to elucidate the specific nature of these relationships.

We speculate that there are multiple potential causal factors that may contribute to circadian misalignment including genetic variations in clock-controlled genes affecting range of entrainment and period of rhythms, mistimed, or irregular zeitgebers (e.g., feeding, exercise, and light patterns), low exposure to zeitgebers (e.g., low light), and hypo- or hypersensitivity to zeitgebers (e.g., circadian light sensitivity). These diverse factors suggest that effective treatment may require personalization of treatment to the specific circadian dysfunction such as phase delay or advance, short or long period, or misalignment (Lewy, 2009; McCarthy et al., 2019). The current evidence suggests that interventions aimed at realigning circadian rhythms may be effective at reducing depressive symptoms, most commonly via phase advance, and particularly in depressed adolescents or young adults who had circadian rhythm delays at baseline (Wescott et al., 2024). In addition, evidence for circadian changes (e.g., phase shift) in response to treatments that do not explicitly target circadian alignment (e.g., ketamine, brexpiprazole) continues to emerge (Moon et al., 2016; Duncan et al., 2017; Krystal et al., 2021). However, to date, there is only preliminary evidence for changes in *internal* circadian alignment following mood disorder treatment (Fang et al., 2021; Krystal et al., 2021). Further longitudinal investigation of circadian misalignment in mental ill-health is essential to establish the nature of the potential causal relationships suggested here and disentangle treatment implications.

In addition to the internal circadian misalignment subgroup, this study identified 2 further subgroups with notable abnormal circadian profiles. Four individuals (~6% of the mood disorder group) presented with sleep-wake patterns indicative of potential *non-24-h sleep-wake disorder* (see Supplementary Materials). Given the drifting nature of these sleep-wake patterns, it is not surprising that abnormal melatonin curves were also observed based on a single assessment scheduled around average sleep times. While the prevalence of non-24-h sleep-wake disorder in sighted individuals is presumed rare (Uchiyama and Lockley, 2015), there is some indication that it is more common among adolescents and young adults (Hayakawa et al., 2005). This disorder has also been reported to be preceded by a psychiatric disorder in 28% of cases, and a further 34% of remaining cases develop major depression thereafter (Hayakawa et al., 2005). Together with the current findings, this highlights the importance of considering non-24-h sleep-wake disorder as a potential circadian disturbance in the assessment and clinical care of young people presenting with mental ill-health and sleep complaints.

In addition, a subgroup of 7 individuals with emerging mood disorders (~10%) had melatonin patterns in which DLMO was not detected across the recording period using the hockey-stick method (see Supplementary Materials). There are several potential explanations for this finding. First, these individuals may be low secretors of melatonin, which falls within the spectrum of normal melatonin production (Burgess and Fogg, 2008). Second, they may have extremely delayed melatonin rhythms, with DLMO occurring beyond 2 h past habitual sleep time. Third, they may be highly sensitive to light: light sensitivity has been reported to vary considerably between individuals (Phillips et al., 2019), and thus the laboratory conditions of this protocol (<30 lux) may have been insufficiently dim to prevent suppression of melatonin; our study was conducted before the extent of melatonin suppression at light levels <10 lux was widely acknowledged, and our protocol followed consensus guidelines for measurement of melatonin in humans (authored by the inventor of the DLMO) which recommended a threshold at <30 lux (Benloucif et al., 2008). This consensus guideline also cautioned that low levels of dim light (<10 lux) that is darker than a subject’s usual light-dark environment may send a “dark pulse” that could inadvertently alter the timing of melatonin onset.

Our study contributes the largest examination to date of the relationships between 3 circadian phase markers in a mood disorder sample. Nevertheless, the results should be interpreted in light of several potentially important methodological limitations. First, the healthy control group was significantly older than the mood disorder group; however, age was controlled for in comparative analyses. Second, the duration of time between actigraphy recording and overnight assessment was up to 30 days due to scheduling constraints and day of the week was not standardized which may have affected sleep-circadian phase associations. Previous research in healthy populations has reported differences in DLMO timing between weekdays and weekends or across weeks of up to 2 h for some individuals (Hasler et al., 2019; Stone et al., 2020; Zerbini et al., 2021); however, to our knowledge, the stability of DLMO or other circadian rhythms has not been studied in mood disorder populations. Given the links between delayed sleep-wake timing, more irregular sleep patterns and later, less stable DLMO (Murray et al., 2019; Watson et al., 2020), as well as irregular sleep patterns and mood disorders (Castiglione-Fontanellaz et al., 2023; Messman et al., 2024), this is an important consideration in interpretation of the current findings. Third, the current protocol was a semi-constant routine with participants remaining seated as much as possible during the circadian assessment, which may be limited in accuracy of circadian estimates compared with other methods (e.g., constant routine, forced desynchrony). Fourth, core body temperature was measured for less than 24 h, and cortisol peak was only available based on 3 samples following habitual wake time, which may not accurately reflect peak timing in all individuals and may be influenced by the cortisol awakening response; we note that these protocol decisions were made to reduce participant burden in a demanding protocol (in the context of a clinical sample with mental disorders). Fifth, we were not powered to examine the potential effects of psychotropic medications on circadian rhythms, which have been reported elsewhere. Sixth, information on other potentially confounding factors such as racial and ethnic identity was not collected. Seventh, our operationalization of the internal circadian misalignment group as ±2 SD of the control mean is an initial attempt to identify extreme abnormalities. Future studies may benefit from more sophisticated methods to operationalize this phenomenon. Finally, while the data here are suggestive of a relationship between circadian factors and mood syndromes in some individuals, the cross-sectional nature of the data precludes any conclusions about causality.

Our study relied on measurement of circadian phase markers under controlled laboratory conditions, which is labor-intensive, costly, and burdensome for participants. A recent wave of digital studies have begun to show the promise of applying mathematical modeling of the circadian system to intensively sampled data on rest, activity, and sleep-wake cycles from wearables (Lim et al., 2024; Song et al., 2024). Several of these studies suggest the capacity to model causal associations among circadian phase and mood states (Song et al., 2024), to predict incident mood episodes (Lim et al., 2024), and to explore the correlates (and consequences) of misalignment among modeled circadian timing and observed sleep-wake parameters (Lee et al., 2024). These innovations have the potential to improve the scalability and feasibility of real-world circadian assessment, which is an area of strong interest in the context of mood disorders (Dunster et al., 2021; Hickie and Crouse, 2024). The application of mathematical and statistical models to wrist-worn wearables may also make it feasible to conduct longitudinal investigations of dynamic associations among circadian misalignment and mental ill-health, of which there are several promising applications for predicting circadian phase and period in healthy individuals (Woelders et al., 2017; Mayer et al., 2024) and night shift workers (Stone et al., 2019). However, the accuracy of these approaches in mood disorder populations remains to be explored, particularly in consideration of the potential impacts of sleep-wake regularity on circadian stability and associated prediction accuracy. Nevertheless, integration of wrist-worn devices (Lee et al., 2024) with other sensors measuring rhythms of temperature (Mason et al., 2024; Shin et al., 2025), heart rate (Bowman et al., 2021; Kim et al., 2023), and light exposure (Crouse et al., 2025; Windred et al., 2024) may allow powerful measurement of circadian rhythmicity in real-world, longitudinal settings.

Together, our findings suggest that multiple distinct circadian abnormalities are observable in young people with mood disorders, and that earlier relative timing of the core body temperature rhythm or later relative DLMO may be key circadian features linked to depressive symptoms. The presence of different abnormalities is consistent with our proposition that a transdiagnostic “circadian depression” phenotype exists (Carpenter et al., 2021), which is driven by circadian dysfunction but may present in different ways depending on the precise nature of the abnormalities and interaction with external factors. Given the complex interplay between internal circadian rhythms, behavior, and the environment, it will be important for future research to measure multiple circadian rhythms longitudinally, particularly in response to treatments, to translate these results into clear clinical implications. Treatments that correct circadian dysfunction (e.g., timed melatonin or melatonin agonists; behavioral regulation of activity, light, and sleep schedules) may have a crucial role in effective personalized treatment.

## Supplemental Material

sj-docx-1-jbr-10.1177_07487304251349408 – Supplemental material for Evidence for Internal Misalignment of Circadian Rhythms in Youth With Emerging Mood Disorders Supplemental material, sj-docx-1-jbr-10.1177_07487304251349408 for Evidence for Internal Misalignment of Circadian Rhythms in Youth With Emerging Mood Disorders by Joanne S. Carpenter, Jacob J. Crouse, Mirim Shin, Emiliana Tonini, Gabrielle Hindmarsh, Zsofi de Haan, Frank Iorfino, Rebecca Robillard, Sharon Naismith, Elizabeth M. Scott and Ian B. Hickie in Journal of Biological Rhythms
