# Supplementary material for: Evidence for Internal Misalignment of Circadian Rhythms in Youth With Emerging Mood Disorders
Source: J Biol Rhythms. 2025 Jul 15;40(5):424–40. doi: 10.1177/07487304251349408 (PMC12426331; doi:10.1177/07487304251349408)
Supplement: sj-docx-1-jbr-10.1177_07487304251349408 – Supplemental material for Evidence for Internal Misalignment of Circadian Rhythms in Youth With Emerging Mood Disorders [file sj-docx-1-jbr-10.1177_07487304251349408.docx]

Supplementary Materials

**Supplementary Figure S1.** Flow diagram showing inclusion and exclusion


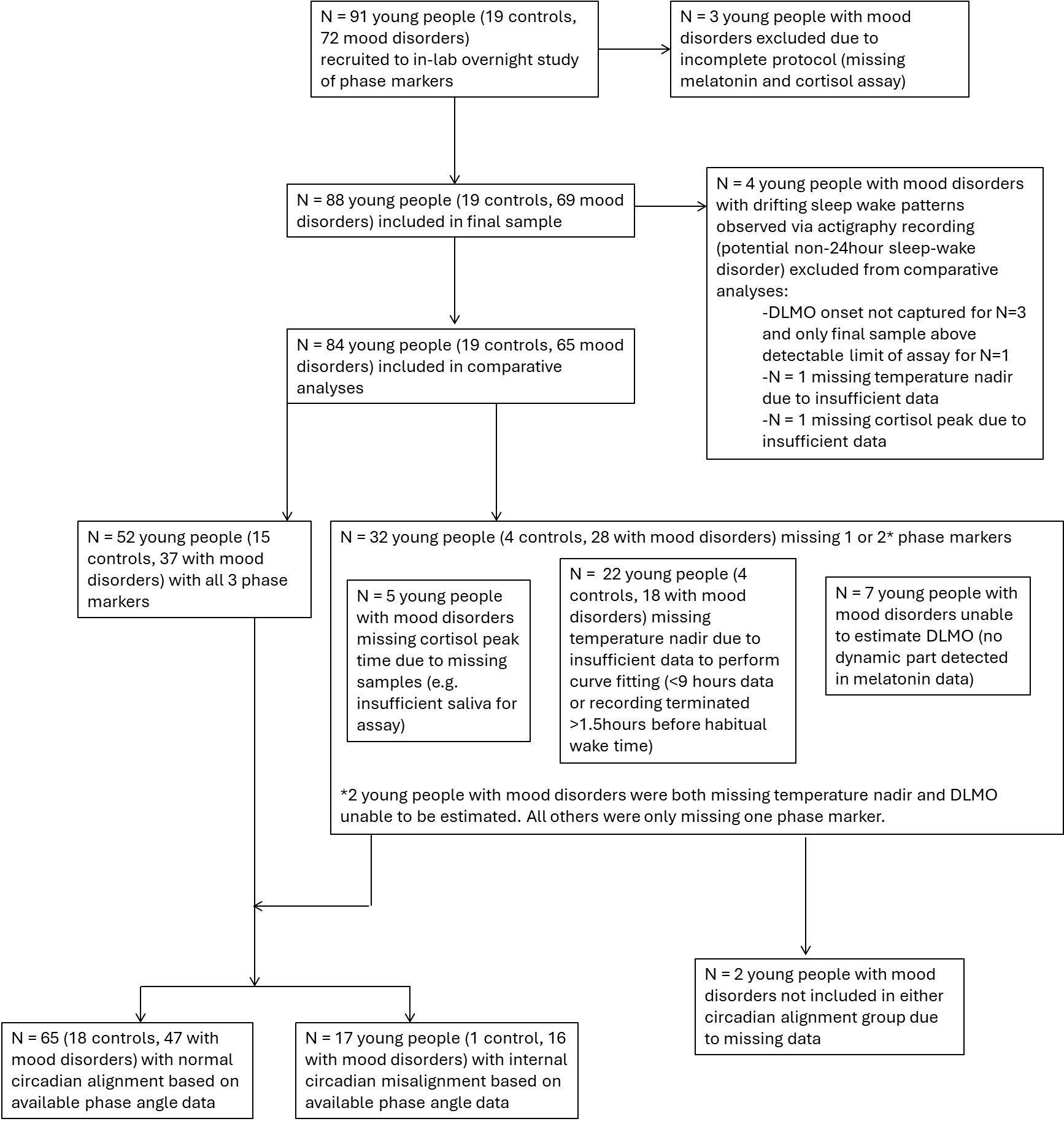


**Supplementary Table S1.** Number of participants with circadian assessments on each day of the week, for control and mood disorder groups.

|  | Control | Mood Disorder |
| --- | --- | --- |
| Monday | 4 | 3 |
| Tuesday | 1 | 13 |
| Wednesday | 2 | 15 |
| Thursday | 5 | 18 |
| Friday | 3 | 13 |
| Saturday | 4 | 7 |
| Sunday | 0 | 0 |

**Supplementary Figure S2.** Actograms showing non-24-hour sleep-wake patterns
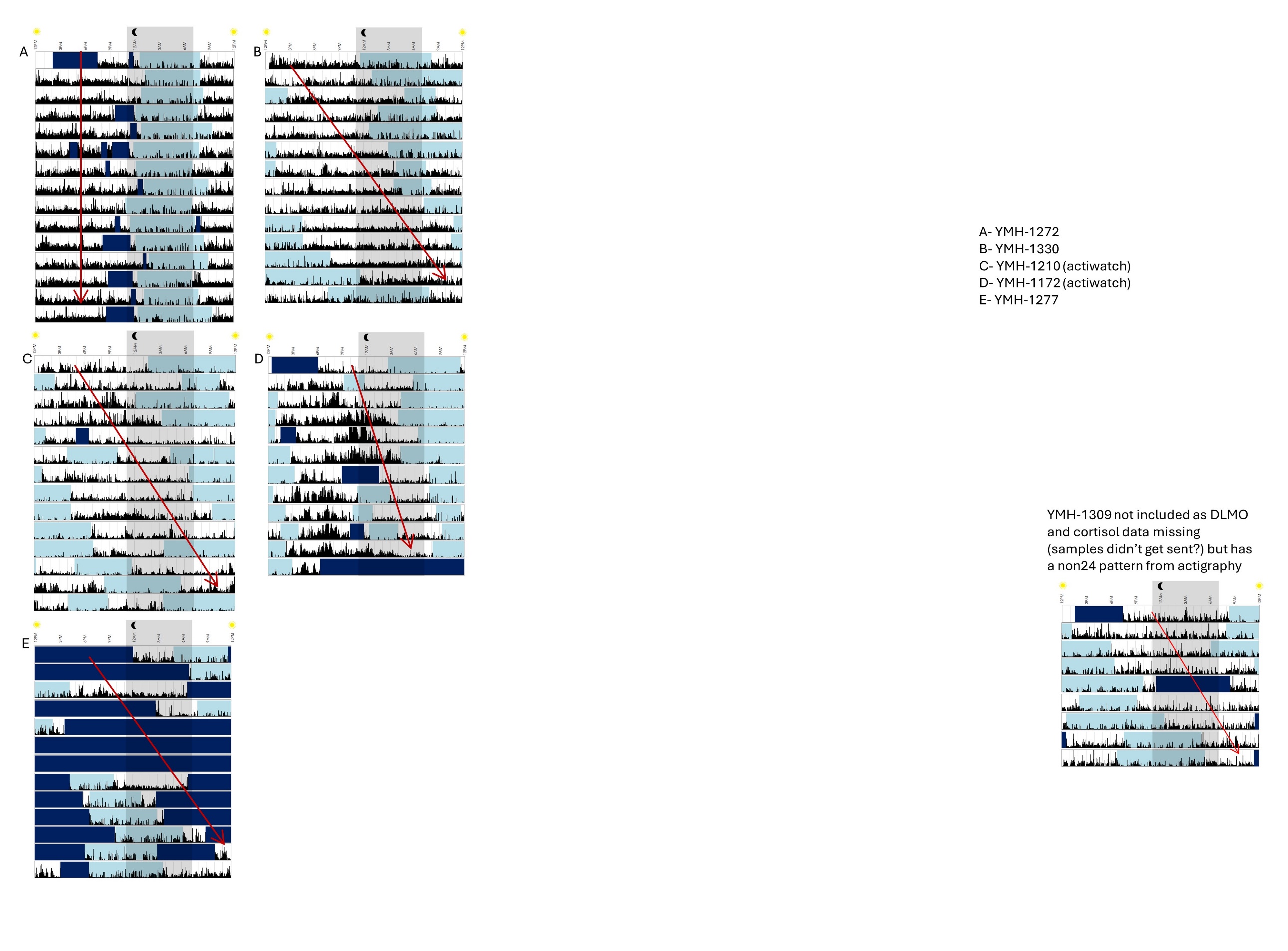


Note: Each row represents one day. Black lines indicate motor activity. Dark blue indicates periods where the actigraphy watch was not worn. Light blue indicates estimates sleep (based on scoring by trained researchers using visual inspection and sleep diaries where available). Grey shaded area indicates ‘ideal’ sleep time from 11pm-7am. Red arrows indicate general drift of sleep-wake timing. Panel A shows an example from a control participant with a typical sleep-wake pattern. Panels B-E show patients with drifting sleep and wake times indicative of potential non-24-hour sleep-wake patterns.

**Supplementary Figure S3.** Salivary melatonin concentrations across the recording period for participants with non-24-hour sleep-wake patterns.


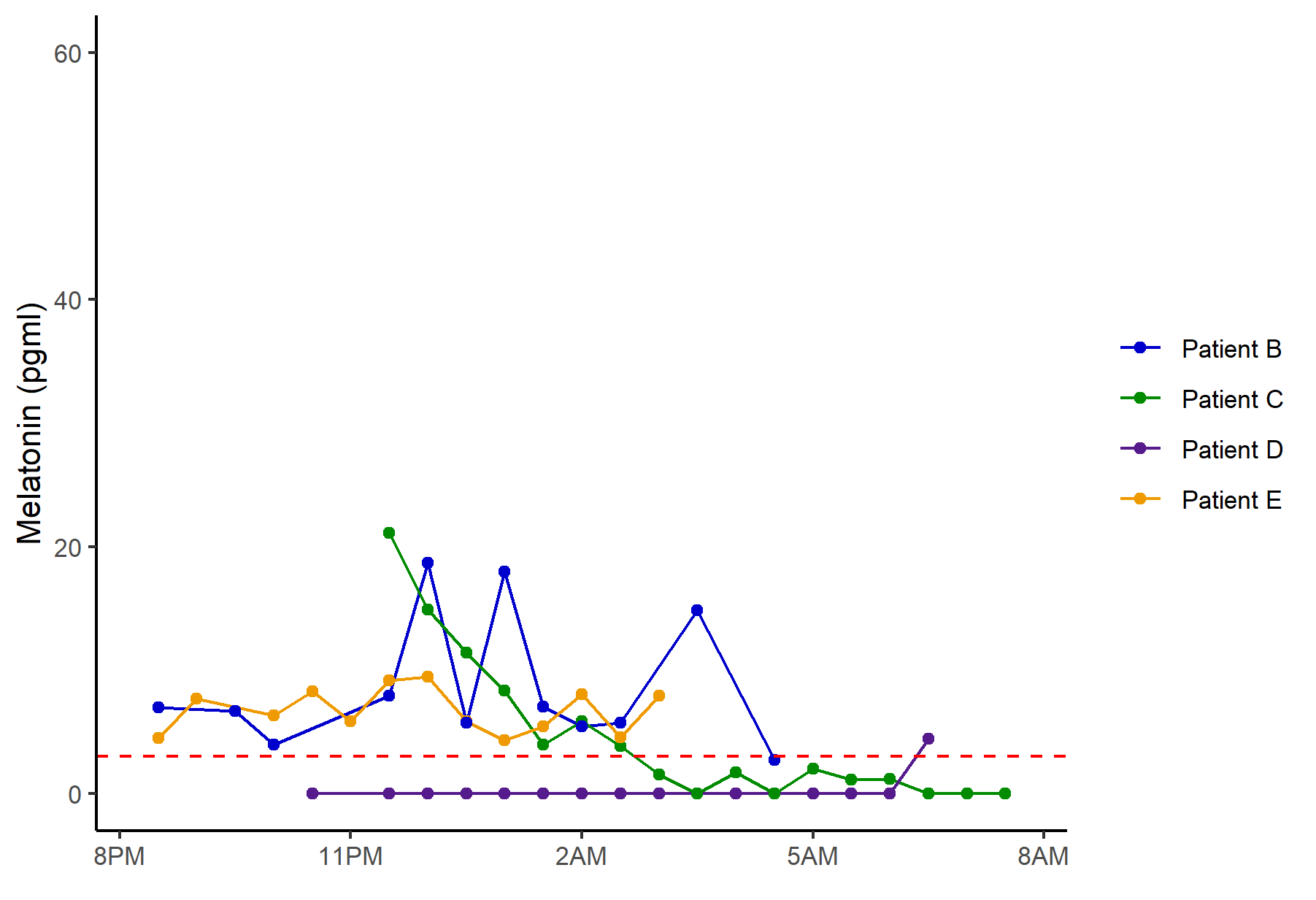


**Supplementary Table S2.** Phase markers for participants with non-24-hour sleep-wake patterns

|  | DLMO time | Core Temperature Nadir time | Cortisol Peak time |
| --- | --- | --- | --- |
| Patient B | - | 9:43 | 12:37 |
| Patient C | - | - | 15:52 |
| Patient D | 6:09 | 11:03 | - |
| Patient E | - | 0:03 | 10:31 |

**Supplementary Figure S4.** Salivary melatonin concentrations across the recording period for participants without a detectable Dim Light Melatonin Onset (N=7)


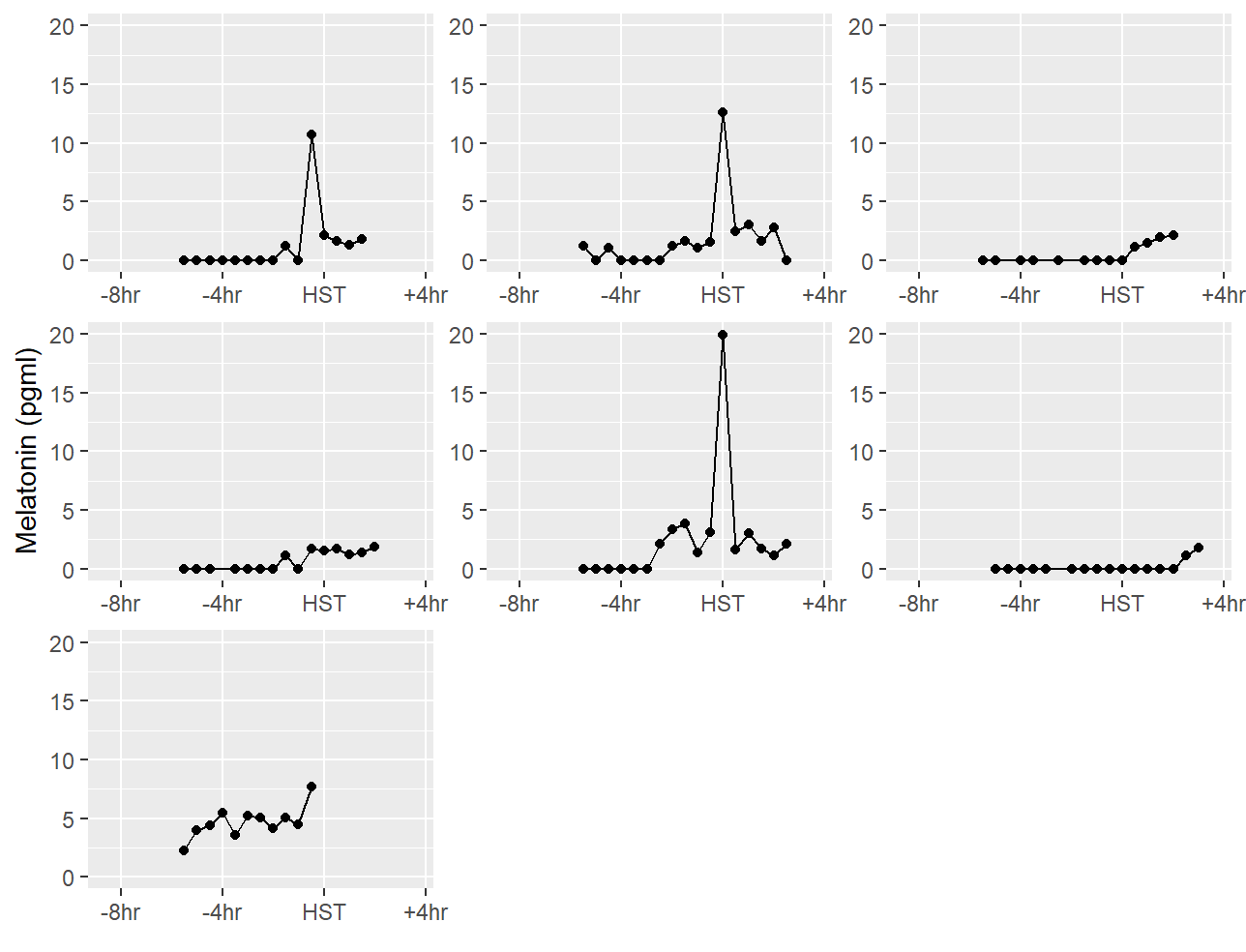


**Supplementary Table S3.** Significant associations (p<0.05) between circadian or phase angle measures and individual HDRS symptom measures controlling for age and sex at birth

| **Predictor Variable** | **Outcome Variable (HDRS item)** | **Intercept** | **F-statistic** | **Slope** | **t-value** | **p-value** |  |
| --- | --- | --- | --- | --- | --- | --- | --- |
| Core Body Temperature Nadir Time | Middle Insomnia | 0.8 | F_(3,35)_=3.16, p=0.037 | -0.21 | -3.01 | 0.005 | ** |
| Core Body Temperature Nadir Time | Anxiety Psychic | 1.47 | F_(3,35)_=4.51, p=0.009 | -0.3 | -3.45 | 0.002 | ** |
| Core Body Temperature Nadir Time | Anxiety Somatic | 0.72 | F_(3,35)_=3.39, p=0.029 | -0.21 | -2.75 | 0.009 | ** |
| Cortisol Peak Time | Late Insomnia | 0.44 | F_(3,46)_=2.98, p=0.041 | -0.14 | -2.55 | 0.014 | * |
| DLMO-Temp Phase Angle | Middle Insomnia | 0.74 | F_(3,29)_=3.76, p=0.022 | -0.28 | -3.23 | 0.003 | ** |
| DLMO-Temp Phase Angle | Work and Activities | 1.49 | F_(3,29)_=4.02, p=0.017 | -0.4 | -3.21 | 0.003 | ** |
| DLMO-Temp Phase Angle | General Somatic | 1.33 | F_(3,29)_=3.49, p=0.028 | -0.23 | -2.89 | 0.007 | ** |
| Temp-Cortisol Phase Angle | Work and Activities | 1.52 | F_(3,29)_=3.06, p=0.044 | 0.44 | 2.81 | 0.009 | ** |
| Temp-Cortisol Phase Angle | Anxiety Psychic | 1.47 | F_(3,29)_=7.22, p<.001 | 0.48 | 4.49 | <0.001 | *** |
| Temp-Cortisol Phase Angle | Weight Loss | -0.01 | F_(3,29)_=4.43, p=0.011 | 0.12 | 3.26 | 0.003 | ** |
| Temp-Sleep Midpoint Phase Angle | Anxiety Psychic | 1.51 | F_(3,34)_=10.73, p<.001 | 0.56 | 5.45 | <0.001 | *** |
| Temp-Sleep Midpoint Phase Angle | Weight Loss | 0 | F_(3,34)_=4.42, p=0.010 | 0.12 | 3.27 | 0.003 | ** |

Multiple linear regression models including age and sex as covariates. Predictor variables were centred across all youth with emerging mood disorders and Female was the reference category for sex. HDRS = Hamilton Depression Rating Scale; DLMO = Dim Light Melatonin Onset; Temp = Core Body Temperature Nadir *p<.05, **p<.01, and ***p<.001 in t-test

**Supplementary Figure S5.** Scatterplots for significant associations between circadian or phase angle measures and individual HDRS symptom measures controlling for age and sex at birth

**
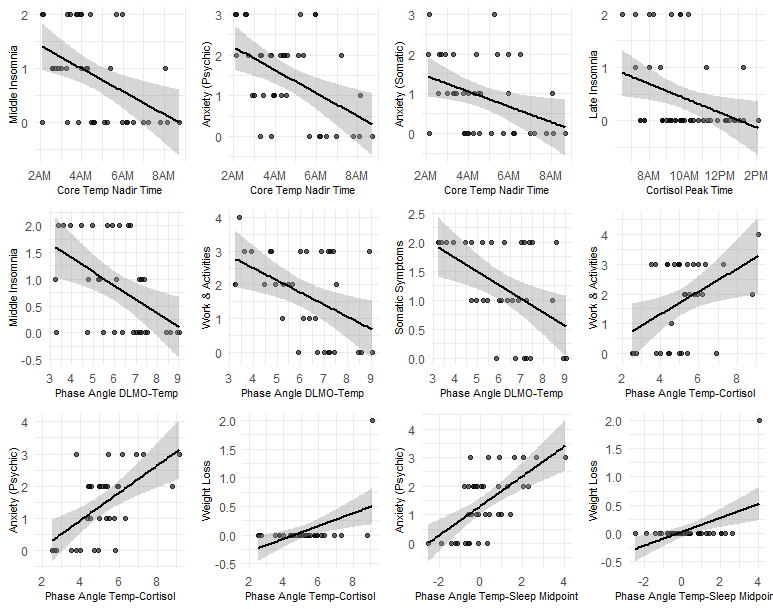
**

HDRS = Hamilton Depression Rating Scale; DLMO = Dim Light Melatonin Onset; Temp = Core Body Temperature Nadir

**Supplementary Results using alternative DLMO calculation with an 3pg/mL threshold.**

Twelve individuals with emerging mood disorders did not reach the 3pg/mL threshold for DLMO across the sampling period.Two of these individuals met criteria for internal misalignment based on Temp-Cortisol Phase Angles and the remaining 10 were not included in either mood disorder group for comparative analyses below. Supplementary Table S4 presents results analogous to Table 2, and Supplementary Table S5 presents results analogous to Table 3 using a 3pg/mL DLMO threshold for all variables involving DLMO and associated group membership definitions.

**Supplementary Table S4.** Associations between circadian (DLMO) and symptom and functioning measures in youth with emerging mood disorders controlling for age and sex at birth using 3pg/mL DLMO threshold

| Outcome Variable | Predictor Variable | Intercept | F-statistic | Slope (B) | t-value | p-value |
| --- | --- | --- | --- | --- | --- | --- |
| SOFAS | DLMO Time | 65.14 | F(3,44)=1.45, p=0.2399 | -0.13 | -0.17 | 0.87 |
|  | DLMO-Temp Phase Angle | 67.52 | F(3,29)=2.68, p=0.0651 | 0 | 0 | 1.00 |
|  | DLMO-Cortisol Phase Angle | 65.27 | F(3,39)=2.18, p=0.1063 | -1.55 | -1.48 | 0.15 |
|  | DLMO-Sleep Midpoint Phase Angle | 65.12 | F(3,44)=1.45, p=0.2426 | 0.05 | 0.05 | 0.96 |
| YMRS | DLMO Time | 2.85 | F(3,37)=0.91, p=0.4434 | -0.54 | -1.46 | 0.15 |
|  | DLMO-Temp Phase Angle | 2.45 | F(3,23)=0.07, p=0.9775 | -0.13 | -0.28 | 0.78 |
|  | DLMO-Cortisol Phase Angle | 2.67 | F(3,32)=0.34, p=0.7948 | 0.2 | 0.36 | 0.72 |
|  | DLMO-Sleep Midpoint Phase Angle | 2.97 | F(3,37)=0.47, p=0.7041 | 0.48 | 0.91 | 0.37 |
| HDRS | DLMO Time | 13.33 | F(3,40)=0.98, p=0.4128 | 0 | 0.01 | 1.00 |
|  | DLMO-Temp Phase Angle | 12.06 | F(3,26)=4.31, p=0.0136 | -2.2 | -3.35 | <.01 |
|  | DLMO-Cortisol Phase Angle | 13.43 | F(3,35)=1.26, p=0.3022 | -0.31 | -0.38 | 0.70 |
|  | DLMO-Sleep Midpoint Phase Angle | 13.17 | F(3,40)=1.16, p=0.3387 | -0.53 | -0.7 | 0.49 |

Multiple linear regression models including age and sex as covariates. Predictor variables were centred across all youth with emerging mood disorders and Female was the reference category for sex. Slopes represent unstandardized regression coefficients. SOFAS SOFAS = Social and Occupational Functioning Assessment Scale; YMRS = Young Mania Rating Scale; HDRS = Hamilton Depression Rating Scale; DLMO = Dim Light Melatonin Onset; Temp = Core Body Temperature Nadir

**Supplementary Table S5.** Comparisons of demographic, sleep, circadian, and clinical measures between groups using 3pg/mL DLMO threshold

|  |  | **Group Mean(SD)** | | | **ANOVA / ANCOVA / χ^2^** | **Pairwise comparisons (Tukey HSD, p values)** | | |
| --- | --- | --- | --- | --- | --- | --- | --- | --- |
|  |  | **Controls Circadian Aligned** | **Mood Disorder Circadian Aligned** | **Mood Disorder Circadian Misaligned** |  | **Control Aligned v Mood Disorder Aligned** | **Control Aligned v Mood Disorder Misaligned** | **Mood Disorder Aligned v Mood Disorder Misaligned** |
|  | N | 19 | 51 | 11 |  |  |  |  |
| **Demographics** | Age (years) | 23.95 (3.60) | 20.63 (3.62) | 19.73 (2.72) | F_(2,78)_=7.43, p=0.001 | <.01** | 0.01* | 0.72 |
|  | Sex (M/F) | 10/9 | 18/33 | 5/6 | χ ^2^_( 2)_=1.84, p=0.398 | - | - | - |
|  | BMI | 23.13 (3.46) | 24.67 (5.78) | 25.04 (3.96) | F_(2,69)_=1.94, p=0.151 | 0.19 | 0.20 | 0.83 |
| **In-Lab Circadian Measures** | DLMO time | 22:07 (1:51) | 22:38 (1:46) | 23:13 (1:40) | F_(2,67)_=1.24, p=0.295 | 0.54 | 0.27 | 0.59 |
|  | Cortisol Peak time | 09:04 (1:15) | 09:50 (1:57) | 10:13 (1:18) | F_(2,70)_=1.47, p=0.236 | 0.42 | 0.22 | 0.61 |
|  | Core Temperature Nadir time | 04:20 (1:32) | 04:43 (1:40) | 04:22 (2:08) | F_(2,57)_=0.3, p=0.74 | 0.80 | 1.00 | 0.83 |
| **Actigraphy Measures** | Sleep Midpoint time | 04:09 (0:55) | 04:52 (1:37) | 04:55 (1:03) | F_(2,74)_=1.65, p=0.2 | 0.20 | 0.32 | 0.97 |
|  | Sleep Duration (minutes) | 436.65(50.48) | 439.82(53.07) | 458.67(37.26) | F_(2,74)_=0.63, p=0.537 | 0.95 | 0.76 | 0.50 |
| **Internal Phase Angles** | DLMO-Temp Phase Angle | 06:14 (1:10) | 05:54 (1:01) | 04:41 (2:57) | F_(2,47)_=2.71, p=0.077 | 0.79 | 0.07 | 0.11 |
|  | Temp-Cortisol Phase Angle | 04:45 (1:16) | 05:00 (1:04) | 05:45 (2:46) | F_(2,51)_=1.24, p=0.299 | 0.96 | 0.32 | 0.31 |
|  | DLMO-Cortisol Phase Angle | 10:56 (1:19) | 11:08 (1:05) | 10:54 (2:17) | F_(2,62)_=0.2, p=0.822 | 1.00 | 0.83 | 0.81 |
| **Circadian/Sleep Phase Angles** | DLMO-Sleep Midpoint Phase Angle | 06:01 (1:20) | 06:07 (1:08) | 05:28 (2:03) | F_(2,66)_=1.02, p=0.367 | 1.00 | 0.41 | 0.35 |
|  | Temp-Sleep Midpoint Phase Angle | -00:19 (1:12) | 00:03 (1:03) | 00:45 (2:09) | F_(2,55)_=1.63, p=0.205 | 0.71 | 0.18 | 0.33 |
|  | Sleep Midpoint-Cortisol Phase Angle | 04:54 (0:48) | 05:00 (0:48) | 05:06 (0:57) | F_(2,70)_=0.16, p=0.852 | 1.00 | 0.86 | 0.85 |
| **Clinical Measures** | Primary Diagnosis (Dep/Bip/Anx/Other) | - | 25/5/9/7 | 5/0/4/0 | χ ^2^_(3)_=4.32, p=0.229 | - | - | - |
|  | SOFAS | - | 63.80 (9.18) | 60.80 ( 6.16) | F_(1,52)_=0.74, p=0.393 | - | - | - |
|  | YMRS | - | 3.75 (4.31) | 0.89 (1.17) | F_(1,45)_=3.66, p=0.062 | - | - | - |
|  | HDRS | - | 14.70 (5.89) | 15.11 (7.15) | F_(1,48)_=0.04, p=0.834 | - | - | - |
| **Medications** | Any Psychiatric Medication N(%) | - | 24/25 | 3/8 | χ ^2^_(1)_=0.95, p=0.331 | - | - | - |
|  | SSRI N(%) | - | 35/14 | 9/2 | χ ^2^_(1)_=0.11, p=0.744 | - | - | - |
|  | SNRI N(%) | - | 43/6 | 10/1 | χ ^2^_(1)_=0, p=1 | - | - | - |
|  | Mood Stabiliser N(%) | - | 44/5 | 9/2 | χ ^2^_(1)_=0.05, p=0.822 | - | - | - |
|  | Stimulant N(%) | - | 48/1 | 11/0 | χ ^2^_(1)_=0, p=1 | - | - | - |
|  | Antipsychotic N(%) | - | 48/1 | 11/0 | χ ^2^_(1)_=0, p=1 | - | - | - |

**Note:** SD = Standard Deviation; BMI = Body Mass Index; DLMO = Dim Light Melatonin Onset; SOFAS = Social and Occupational Functioning Assessment Scale; YMRS = Young Mania Rating Scale; HDRS = Hamilton Depression Rating Scale; SSRI = Selective Serotonin Reuptake Inhibitor; SNRI Serotonin and Norepinephrine Reuptake Inhibitor; ANOVA = Analysis of Variance (for demographic variables only); ANCOVA = Analysis of Covariance controlling for Age and Sex (for all other continuous variables); Phase angles and time variables are presented as hours and minutes. *p<.05 and **p<.01 in pairwise tests
